# DTI Abnormalities Related to Glioblastoma: A Prospective Comparative Study with Metastasis and Healthy Subjects

**DOI:** 10.3390/curroncol29040230

**Published:** 2022-04-16

**Authors:** Youssef El Ouadih, Bruno Pereira, Julian Biau, Béatrice Claise, Rémi Chaix, Pierre Verrelle, Toufik Khalil, Xavier Durando, Jean-Jacques Lemaire

**Affiliations:** 1 Service de Neurochirurgie, Institut Pascal, Université Clermont Auvergne, Clermont Auvergne INP, CHU Clermont-Ferrand, CNRS, F-63000 Clermont-Ferrand, France; youssef.el_ouadih@ghu-paris.fr (Y.E.O.); bclaise@chu-clermontferrand.fr (B.C.); rchaix@chu-clermontferrand.fr (R.C.); tkhalil@chu-clermontferrand.fr (T.K.); 2 GHU Psychiatrie et Neurosciences, Neurochirurgie, F-75014 Paris, France; 3 Delegation for Clinical Research and Innovation, Université Clermont Auvergne, CHU Clermont-Ferrand, F-63000 Clermont-Ferrand, France; bpereira@chu-clermontferrand.fr; 4 Department of Radiation Oncology, Université Clermont Auvergne, Centre Jean Perrin, F-63000 Clermont-Ferrand, France; julian.biau@clermont.unicancer.fr (J.B.); pierre.verrelle@curie.fr (P.V.); xavier.durando@cjp.fr (X.D.); 5 Service de Radiothérapie, Institut Curie, F-75005 Paris, France

**Keywords:** glioblastoma, metastasis, infiltration, MRI, DTI, tractography, brain adjacent to tumor, diffusion, anisotropy, biomarker

## Abstract

(1) Background: Glioblastoma multiforme (GBM) shows complex mechanisms of spreading of the tumor cells, up to remote areas, and little is still known of these mechanisms, thus we focused on MRI abnormalities observable in the tumor and the brain adjacent to the lesion, up to the contralateral hemisphere, with a special interest on tensor diffusion imaging informing on white matter architecture; (2) Material and Methods: volumes, macroscopic volume (MV), brain-adjacent-tumor (BAT) volume and abnormal color-coded DTI volume (aCCV), and region-of-interest samples (probe volumes, ipsi, and contra lateral to the lesion), with their MRI characteristics, apparent diffusion coefficient (ADC), fractional anisotropy (FA) values, and number of fibers (DTI fiber tracking) were analyzed in patients suffering GBM (*n* = 15) and metastasis (*n* = 9), and healthy subjects (*n* = 15), using ad hoc statistical methods (type I error = 5%) (3) Results: GBM volumes were larger than metastasis volumes, aCCV being larger in GBM and BAT ADC was higher in metastasis, ADC decreased centripetally in metastasis, FA increased centripetally either in GBM or metastasis, MV and BAT FA values were higher in GBM, ipsi FA values of GBM ROIs were higher than those of metastasis, and the GBM ipsi number of fibers was higher than the GBM contra number of fibers; (4) Conclusions: The MV, BAT and especially the aCCV, as well as their related water diffusion characteristics, could be useful biomarkers in oncology and functional oncology.

## 1. Introduction

The treatment of high grade glioma, and particularly resection, is still challenging due to its well-known infiltrative nature [1,2,3,4] and the presence of inconspicuous, remote, tumor cells [5]. A multicellular network relying on microtubes could support the invasion and proliferation characteristics of this widespread disease [6]. Pioneering works have also shown that glioblastoma multiforme (GBM) growth appears typically along white matter (WM) tracts [7]. Phenotypically, the macroscopic tumor volume is still accepted, notably for GBM, as the volume limited by the contrast enhanced boundary on magnetic resonance imaging (MRI) [8], which serves to define the gross tumor volume for radiotherapy [9], and to which is added the brain adjacent to tumor (BAT) [10]. In GBM, MRI spectroscopy features of BAT, high choline/N-acetyl-aspartate ratio and choline/creatinine ratio, argue for tumor invasion [11]. Histologically the tumor cell density decreases up to several centimeters from the macroscopic tumor volume [12]. In current clinical practice, the BAT can be referred to as the region adjacent to the gross tumor volume and which contains signal abnormalities on T2-weighted and fluid-attenuated inversion recovery (FLAIR) sequences, and the limit of the BAT serves as the definition of the clinical target volume (CTV) in radiotherapy [9]. Diffusion weighted imaging (DWI) and diffusion tensor imaging (DTI) also revealed signal abnormalities in the BAT, and the nature of lesions of white matter fascicles is multiple such as infiltration, disruption, cellular and vasogenic oedema, and displacement [13,14]. Analysis of metrics of diffusion parameters in the BAT have shown: (1) fractional anisotropy (FA) of high grade gliomas is higher than for metastasis and conversely for the mean diffusivity (MD) [15]; nevertheless, it was also found that FA is lower in BAT than outside and inversely correlated to the degree of cell invasion [16]; (2) diffusion values (apparent diffusion coefficient, ADC; signal intensity on DWI) in the BAT are high in GBM, and higher than for metastasis [17]; (3) relative anisotropy in the normal T2-weighted zone is reduced and could be more specific for GBM than for metastasis and low grade glioma [18]; (4) in high grade gliomas, FA decreases and ADC increases in BAT, which is sometimes apparently normal in routine MRI [19]; (5) FA values are reduced in apparently normal and pathologic corpus callosum of patients with GBM [20]. The study of DTI fiber density in grades II and III gliomas have shown that high fiber density values are inversely correlated with the tumor cell count and infiltration, in the macroscopic tumor volume but not at its border [21]. In the GBM BAT, the DTI fiber density seems correlated with the mean FA value, which is reduced relative to the homologous contralateral region [22]. Thus, the detailed MRI analysis of the BAT is of upmost importance, as it enables the detection of subtle abnormalities, which are not specific to precise lesioning mechanisms. The growing interest in artificial intelligence methods enabling the detection of tumors on medical imaging, and the importance of optimal topographically directed molecular analysis of tissue and of planning and follow-up strategies of treatments, impose the possibility of refining our understanding of the macroscopic phenotypic definition of GBM and its invasiveness [23,24,25,26].

In that respect, we aimed to study prospectively diffusion related parameters of MRI datasets of two series of patients suffering GBM and metastasis, and of a control group of healthy subjects. We compared the values of parameters in the macroscopic tumor volume (MV), the BAT volume, and the abnormal DTI brain volume around the BAT. The abnormal DTI brain volume was determined on color-coded DTI maps that reveal the microarchitecture of the white matter and was named aCCV (abnormal color-coded volume). The aCCV appears “normal” in conventional imaging that shows the MV and the BAT; this latter is still frequently caricatured as “oedema volume” in the clinical field. The metastatic lesions were chosen for comparison with GBM because most are restrained with millimetric invasion [27,28], and as such is frequently used as the opposite model of infiltration of gliomas (see above).

## 2. Materials and Methods

### 2.1. Population

Thirty-nine subjects were enrolled (monocentric) from March 2015 to December 2018 (Institutional Review Board approval). The inclusion criteria were: age > 18 years; no history of inflammatory or degenerative brain disease; patients with unilateral supra-tentorial tumor and a Karnofsky index > 60, and up to 3 unilateral supra-tentorial metastases. All subjects underwent an MRI exam. They all had standard corticoid and anti-epileptic oral treatment since their admission. Two patients were excluded after MRI protocol showing more than three metastases. The histopathological diagnosis after oncologic multidisciplinary meeting, specified the lesions, GBM or metastasis, enabling the inclusion of 24 patients: 15 cases of GBM, mean age = 66.6 ± 9.2 years, 9 males; 9 cases of metastases, mean age = 63.7 ± 11.0 years, 5 males. The control group consisted in 15 healthy subjects with a mean age of 62.7 ± 6.4 years, 7 males comparable to the patients. Demographics (15 GBM + 9 metastasis; 15 healthy subjects) and tumoral characteristics (24 patients) are in Table 1.

### 2.2. MRI

All MRI were performed on 3-Tesla machines (Magnetom 3T, Siemens AG, Munich, Germany; Discovery MR750, General Electric, Milwaukee, WI, USA) with a 32-channel head coil following a routine clinical protocol: T1-weigthed, T1-weighetd enhanced (except for healthy subject), T2-weigthed and FLAIR sequences, and a 20-direction DTI sequence. The images characteristics were: field of view = 240 mm; matrix 512 × 512; 3D T1, repetition time (TR) = 8.8 ms, echo time (TE) = 3.5 ms, voxel size = 0.469 mm side, slice thickness = 1.4 mm; axial T2 axial, Spin Echo, TR = 9000 ms, TE = 80 ms, voxel size = 0.469 mm side, slice thickness = 4 mm; sagittal 3D FLAIR, TR = 9000 ms, TE = 141 ms, voxel size = 0.5 mm side, slice thickness = 1 mm; DTI, TR = 7000 ms, TE = 81 ms, matrix 256 × 256, voxel size = 1 mm side, slice thickness = 3.5 mm, b = 1000 s/mm^2^. Color-coded maps were built (Iplan Stereotaxy 3.0.2; BrainLab; München, Germany) from DTI data sets.

### 2.3. MRI Objects

MRI objects consisted in three volumes, manually contoured (slice by slice) and three ROIs positioned manually. This was realized after co-registration of image datasets and alignment along the anterior—posterior commissure (AC-PC) plan easing the visual comparisons (Iplan Stereotaxy 3.0.2; BrainLab; idem, Germany).

The tumoral lesion was segregated into three volumes, MV, BAT, and aCCV. The MV was defined on the 3D T1-enhanced sequence, the BAT on the 3D FLAIR and B0 diffusion sequences (hypersignal; excluding MV), and the aCCV on the color-coded DTI maps (excluding BAT). The aCCV was defined by comparison between the ipsilateral (where the lesion was) and the contralateral hemispheres, as well as the healthy subject data sets if necessary.

**Figure 1:**
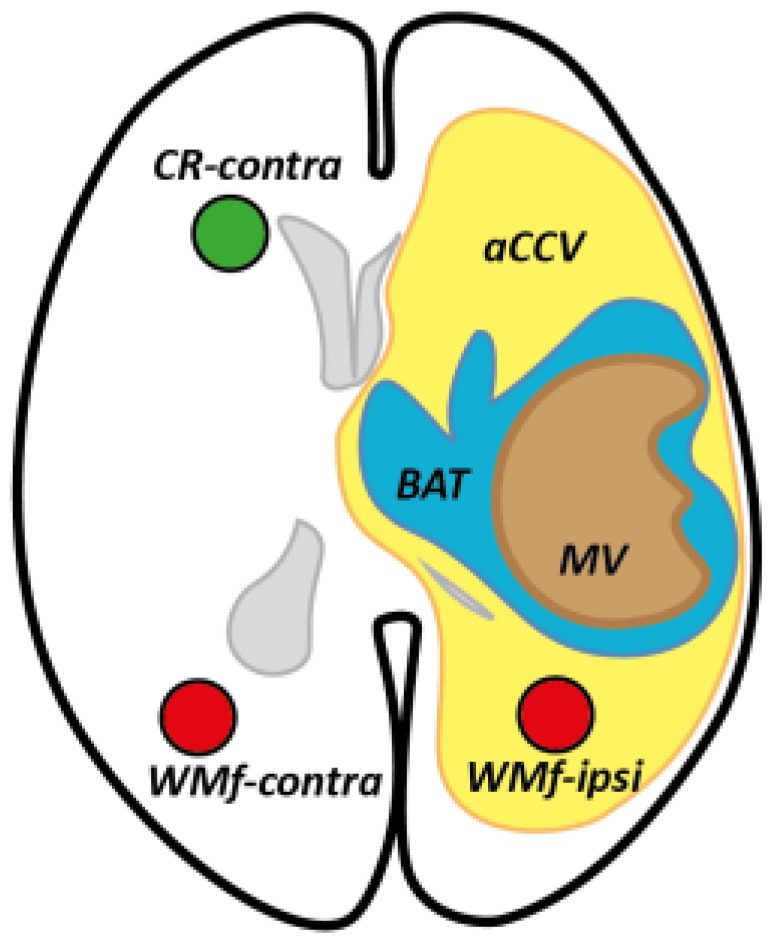
MRI objects schematized on a simplified axial MRI slice (ventricles in gray): (1) volumes, the macroscopic volume (MV), the brain adjacent to tumor (BAT) and the abnormal color-coded volume (aCCV); (2) ROIs in the corona radiata (CR-contra) and white matter fascicles (WMf), ispi- and contra- lateral to the lesion.

**Figure 2:**
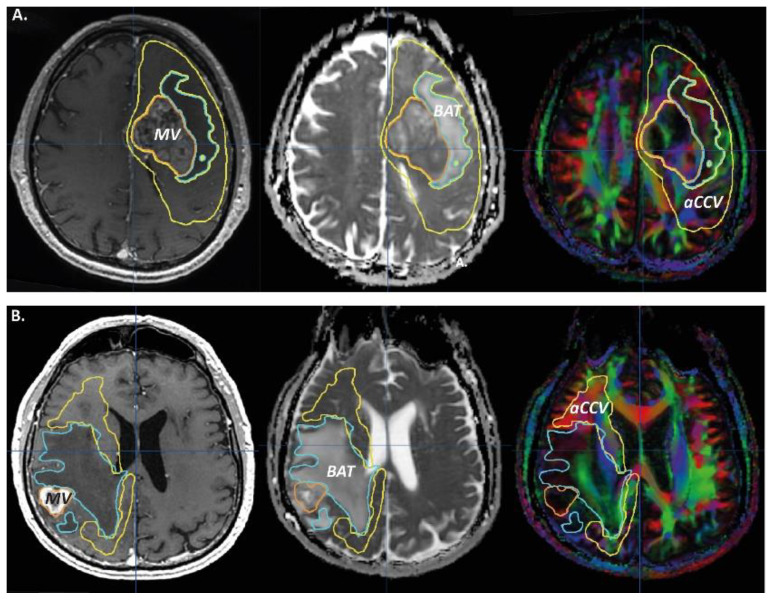
Manual contouring of the macroscopic volume (MV, orange), the BAT volume (blue) and the abnormal color-coded volume (aCCV, yellow) on axial MRI of GBM ((**A**); patient 11; left GBM) and metastasis ((**B**); patient 3; right metastasis) cases; left column, 3D T1-enhanced sequence; intermediate column, B0 diffusion weighted sequence; right column, color-coded DTI map.

**Figure 3:**
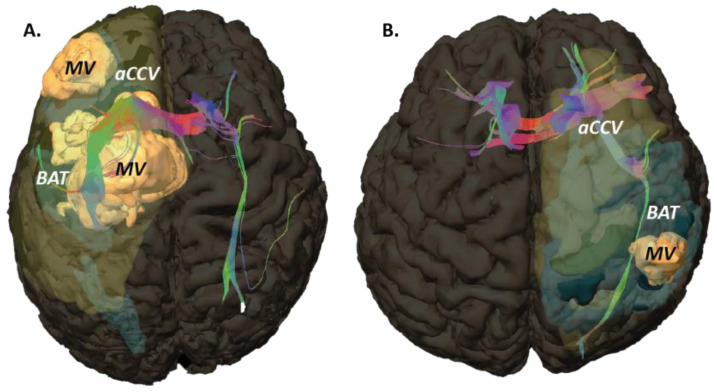
3D rendering (superior view) of the macroscopic volume (MV, orange), the BAT volume (light blue) and the abnormal color-coded volume (aCCV, light yellow) within the brain volume (same patients of the Figure 2; (**A**), GBM; (**B**), metastasis); fascicles computed (fiber tracking DTI) from WMf-ipsi and WMf-contra are embedded.

We also placed region-of-interest samples, ROIs (diameter, 10 mm; thickness, 3.5 mm), in the contralateral frontal corona radiata, 10 mm above the lateral ventricle (CR-contra), and in fascicles of the white matter of the aCCV where we observed the brightest abnormal hue (highest abnormality; WMf-ipsi) and in the contralateral corresponding region (that visually fitted at best; WMf-contra). In the healthy subjects, we placed CR ROIs bilaterally (CR-right; CR-left). The MRI objects are summarized in Figure 1, Figure 2 and Figure 3.

We computed the volumes of MV, BAT, and aCCV (cm^3^), the mean apparent diffusion coefficient (ADC; 10^−3^ × mm^2^/s; the higher value, the higher water-molecule diffusion) and the fractional anisotropy (FA; from 0, isotropic diffusion, to 1, anisotropic diffusion) values of the MRI objects; volumes and ROIs of the 24 patients; only the ROIs for the 15 healthy subjects. For the GBM group, we also calculated mean ADC values of a sample of patients (*n* = 5 out of 15; GBM5) who were explored with the same 3T MRI machine (Discovery MR750, General Electric, Milwaukee, WI, USA) as the metastasis group and the healthy subjects, in an attempt to limit a potential but limited impact of the machine on this parameter [29].

Fiber tracking was realized using fiber assignment by continuous tracking (FACT; FA threshold ≥ 0.2; length of fibers ≥ 50 mm) from seeds in WMf ROIs, and fiber count was extracted from the volume wrapping of tracked fibers (Iplan Stereotaxy 3.0.2; BrainLab; idem, Germany). We noticed the mean FA value and the number of fibers.

### 2.4. Data Analysis

We compared MV, BAT, and aCCV volumes, and mean ADC and FA values, between GBM and metastasis. We compared mean ADC, mean FA, and number of fibers between GBM and metastasis, according to ROIs. The mean ADC and the mean FA values of CR-right and CR-left of healthy subjects, were compared with CR-contra of GBM and metastasis. We compared MV, BAT, aCCV values (volume, mean ADC, and mean FA) of lesions, as well as ipsi vs. contra values of ROIs (mean ADC, mean FA, and number of fibers; WMf and CR ROIs) in GBM and metastasis.

Continuous data (i.e., volumes, ADC, FA, number of fibers, MV, BAT, aCCV, ipsi and CR-contra, WMf) were expressed as mean and standard deviation according to statistical distribution. The assumption of normality was assessed using the Shapiro–Wilk test. The comparisons between independent groups (GBM vs. metastasis vs. HS) were carried out using ANOVA or Kruskal-Wallis test when the assumptions of the ANOVA were not met. The homoscedasticity assumption was studied by using Bartlett’s test. When appropriate (omnibus *p*-value less than 0.05), post-hoc tests for two by two comparisons were performed to take into account multiple comparisons, respectively, Tukey-Kramer after ANOVA and Dunn after Kruskal-Wallis. For comparisons concerning correlated data (i.e., when several measures for a same patient were collected: volumes and ROIs), random-effects models (i.e., mixed linear regression) were used to model between and within subject variability (as random-effect). A Sidak’s type I error correction was applied for multiple comparisons. The normality of residuals from these models was studied as aforementioned. When appropriate, a logarithmic transformation was applied. Statistical analyses were performed using Stata software, Version 15 (StataCorp, College Station, TX, USA). The tests were two-sided with a type I error set at 5%

## 3. Results

The results are synthesized in Table 2.

### 3.1. Tumoral Related Volumes

The mean MV and aCCV of GBM were larger than those of metastasis. In GBM, the mean MV and BAT were comparable (*p* = 0.135) but the mean aCCV was larger than MV (*p* < 0.001) and BAT (*p* < 0.001). In metastasis, the mean BAT was larger than MV (*p* = 0.001) but the other volumes were comparable (aCCV vs. BAT, *p* = 0.100; aCCV vs. MV, 0.107).

The mean ADC value of BAT was higher in metastasis than in GBM5. In GBM5, the mean ADC value of BAT and aCCV were lower than MV (respectively, *p* = 0.01 and <0.001), but the mean ADC values of aCCV and BAT were comparable (*p* = 0.4566). In metastasis, the mean ADC value of aCCV was lower than MV (*p* < 0.001) and BAT (*p* < 0.001).

The mean FA values of MV and BAT were higher in GBM than in metastasis. In GBM and metastasis, the mean FA values increased from MV to BAT then aCCV (*p* < 0.001 for MV vs. BAT, BAT vs. aCCV and MV vs. aCCV).

### 3.2. ROIs

The mean ADC and FA CR-ROIs values of healthy controls were comparable between the right and left hemispheres (*p* = 0.99), as well as CR-contra of GBM and metastasis. In GBM and metastasis, the mean ADC values of WMf-ipsi were comparable to WMf-contra and to CR-contra (GBM, WMf-contra, *p* = 0.408; GBM, CR-contra, *p* = 0.310; metastasis, WMf-contra, *p* = 0.257; metastasis, CR-contra, *p* = 0.286).

In GBM, the mean FA values of WMf-ipsi were higher than WMf-contra (*p* = 0.003) and WMf-contra was comparable to CR-contra (*p* = 0.834). In metastasis, the mean FA values of WMf-ipsi were comparable to WMf-contra (*p* = 0.319) and to CR-contra (*p* = 0.209). The number of fibers (ipsi and contra) were comparable between GBM and metastasis. In GBM, the number of fibers of the WMf-ipsi was higher than that of the WMf-contra (*p* = 0.039), whereas it was comparable in metastasis (*p* = 0.086).

## 4. Discussion

**Figure 4:**
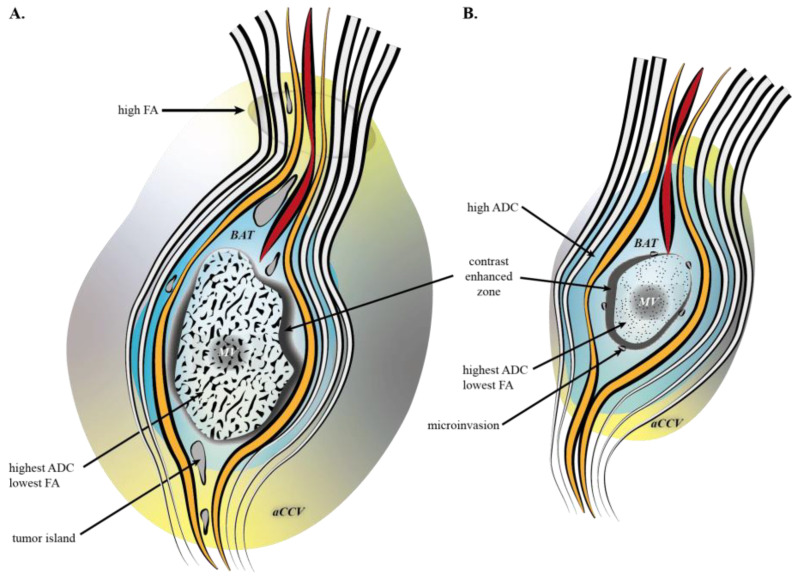
Artistic drawing of MRI characteristics of GBM (**A**), and metastasis (**B**): the highest ADC value is in MV; the BAT ADC value is higher in metastasis; a high FA value is in GBM aCCV; fascicles are depicted as white/black ribbons, structurally lesioned in red, and functionally harmed in orange.

Based on our MRI-tissue-volume analysis, in GBM we found that aCCV volume was larger than MV and BAT volumes, which were larger than those of metastasis. In metastasis, BAT volume was comparable to aCCV, and larger than MV. Thus, it is as if the GBM MV is larger and associated with two adjacent coronas, BAT and beyond aCCV, whereas metastasis has smaller MV and is surrounded by relatively similar BAT and aCCV volumes. The wider volumetric extension and larger volumes in GBM, relative to metastasis, is consistent with sparse data [30,31,32]. We also found that proton-diffusion features were common to GBM and metastasis: the mean-ADC value decreases, centripetally, progressively from MV to aCCV, and conversely for the mean-FA value, altogether with the centripetal lowering of lesioning processes. The mean ADC values of GBM and metastasis were above normal values, ranging from 0.71 to 0.90 × 10^−3^ mm^2^/s [33,34,35,36] that include the values observed in our group of healthy subjects (CR, ROIs). They reached almost normal values in the aCCV. The disorganization of tissue architecture, i.e., of the fascicular organization of fiber bundles, seems less severe in BAT of GBM. Indeed, in GBM, MV and BAT FA values were lower than in metastasis, and BAT mean-FA values were about 0.20, which is a normal threshold value usually considered for fiber tracking of fascicles in white matter [37,38,39]. Normal FA values range from 0.4 to 0.8 [40,41], and depend on the white matter architecture, i.e., the 3D organization of fascicles and the conservation of axonal membranes [42], and at a lesser extent on an MRI machine [43]. Thus, the core of the structural disorganization is maximal (highest ADC; lower FA) within the MV, where the necrosis developed, and which is surrounded by an intense immune reaction [44] and noticeably the angiogenesis identified on CT-Scan, MRI, and ultrasound [45,46]. In GBM, the diffusion decreased between MV and either BAT or aCCV, which had comparable mean ADC values likely because of the infiltrative nature of the lesion [16]. In metastasis, we found the centrifugal gradient of reduction of ADC values, from MV to BAT then aCCV. In BAT, the mean ADC value was higher in metastasis than in GBM, in line with another work [47], showing a higher water diffusivity within the close vicinity of macroscopic tumoral volume where metastasis develop a complex peritumoral edema [48,49]. The Figure 4 synthetizes results.

ROIs data showed that the white matter contralateral to the lesion (CR-contra), either in GBM or metastasis, had normal ADC and FA values (CR-R&L). The mean ADC values, contra- and ispsi- lateral, were also comparable, either in GBM or metastasis. Yet, we found a specific anisotropic pattern of GBM, i.e., ipsilateral high anisotropy coupled with a high number of fiber bundles, while the numbers of fibers were comparable between GBM and metastasis. These findings rely on precise positioning of topographically comparable regions, enabling a fair comparison between mean-FA values. Altogether, a high number of fiber and high mean FA, should reflect a densification of axons, compacted (Figure 3), with reorientation of bundles. This could also reflect the presence of long distance invasion and/or its consequence, such as fascicle disorganization or edema, knowing that water diffusion relies on the size of extracellular space but also on water channels [50,51]. The intime mechanisms must be further determined. This particular aspect, i.e., focal high-anisotropy, completes patterns with normal or low anisotropy, reorientation of fibers and diffusion modifications [52,53]. The impact of such modifications during the exploration of brain function, i.e., the functional neuro-oncology, notably intraoperatively in the vicinity of glioma, regardless of the method such as electrostimulation during awake surgery and/or fiber tracking navigation under general anesthesia, must be explored. New fiber tracking techniques could be helpful to refine the analysis [54]. Interestingly, high ADC and low FA values seem reversible following radiation therapy combined with gefitinib or tipifarnib in high grade glioma of the brainstem in pediatric patients [55].

Globally our results detail the information harvested by the analysis of normal-appearing white matter (NAWM) reporting low FA and ADC values, bearing in mind that NAWM is less restrictive than aCCV as it includes the whole brain with a normal aspect on contrast-enhanced-T1 and FLAIR [13,56], i.e., the whole brain around the BAT.

## 5. Conclusions

In conclusion, our findings highlight structural abnormalities revealed by DTI, which seems a promising in vivo modality of brain tumor exploration [57], accompanied by a careful interpretation of the information provided [58]. The MV, BAT, and especially the aCCV based on normalized color-coded maps, could be useful biomarkers in oncology and functional oncology. For example, they could guide the determination of spots for biopsies and study of molecular markers [59], and refine the individualization of CTV [60]. In the clinical field, before biopsy, the MRI volume features could also assist in distinguishing between GBM and metastasis, which is sometimes difficult. Further progress in radiomics [24,61,62,63] and data analysis [64] should enable the optimization of the analysis of this information. The presence of focal high-anisotropy areas in the aCCV opens a new window on the GBM disease that impacts, functionally and structurally, remote brain areas.

## Figures and Tables

**Table 1:** Characteristics of glioblastoma (GBM) and metastasis groups of patients (biopsy = stereotactic biopsy; resection = surgical tumor resection).

**GBM**				
**Age**	**Gender**	**Location**	**Symptoms**	**Tumor Sample**
70	M	occipital (left)	memory disorder	biopsy
70	M	anterior cingulate (left)	cognitive disorder, contralateral motor paresis	biopsy
67	M	fronto-temporo-insular (left)	frontal syndrome, aphasia, contralateral motor paresis	biopsy
71	M	fronto-callosal (right)	frontal syndrome	resection
63	M	temporal (left)	headache, aphasia, contralateral motor paresis	resection
67	F	occipital (right)	headache, lateral homonym hemianopsia	biopsy
66	F	temporo-insular (right)	contralateral motor paresis	resection
55	M	temporo-insular (left)	behavior disorder, aphasia, contralateral facial paresis	biopsy
61	F	anterior cingulate (right)	contralateral motor paresis	biopsy
65	M	temporal (right)	headache, contralateral facial paresis	resection
78	M	fronto-temporo-insular (left)	frontal syndrome, aphasia, contralateral motor paresis	biopsy
75	F	occipital (right)	headache, lateral homonym hemianopsia	resection
49	F	frontal (left)	aphasia	resection
85	M	temporo-insular (left)	frontal syndrome, aphasia	biopsy
57	F	frontal (right)	memory disorder, contralateral facial paresis	resection
**Metastasis**				
**Age**	**Gender**	**Location**	**Symptoms (Cancer)**	**Tumor Sample**
78	F	frontal (left)	inaugural (lung)	biopsy
55	M	paraventricular trigone (left)	headache (neuroendocrine)	resection
71	M	parietal (right)	contralateral motor paresis (kidney)	resection
70	F	temporal and parietal (right)	contralateral motor paresis (lung)	resection
64	F	precentral (left)	contralateral motor paresis (lung)	resection
43	M	precentral (right)	contralateral motor paresis (lung)	resection
59	M	temporal (left)	inaugural seizure (lung)	resection
75	M	frontal (left)	headache, contralateral motor paresis (lung)	resection
59	F	frontal (left)	aphasia (anal)	resection

**Table 2:** Values of MRI-object related parameters of the glioblastoma (GBM; GBM5 * see text), metastasis, and healthy subject (HS) groups.

MRI Objects			GBM (GBM5 *)		Metastasis		HS		Difference
			Mean	SD	Mean	SD	Mean	SD	*p* Value
Volumes	volume (cm^3^)	MV	47.12	28.44	12.96	17.61	n.a.	n.a.	0.0032
		BAT	70.65	42.01	75.98	73.93	n.a.	n.a.	0.6983
		aCCV	176,49	74,21	44.80	44.73	n.a.	n.a.	0.0006
	mean ADC value (10^−3^ × mm²/s)	MV	1.726 *	0.527 *	1.539	0.287	n.a.	n.a.	0.4634
		BAT	1.093 *	0.527 *	1.504	0.158	n.a.	n.a.	0.0196
		aCCV	0.910 *	0.088 *	0.920	0.103	n.a.	n.a.	0.9469
	mean FA value	MV	0.135	0.046	0.089	0.017	n.a.	n.a.	0.0026
		BAT	0.206	0.056	0.154	0.026	n.a.	n.a.	0.0157
		aCCV	0.315	0.052	0.324	0.062	n.a.	n.a.	0.6123
ROIs	mean ADC value (10^−3^ × mm²/s)	CR-contra	0.708 *	0.343 *	0.722	0.220	n.a.	n.a.	0.2527 (vs. CR-R_L)
		WMf-ipsi	0.840 *	0.071 *	0.817	0.056	n.a.	n.a.	0.5485
		WMf-contra	0.849 *	0.059 *	0.797	0.046	n.a.	n.a.	0.1615
		CR-right (CR-R)	n.a.	n.a.	n.a.	n.a.	0.801	0.032	n.a.
		CR-left (CR-L)	n.a.	n.a.	n.a.	n.a.	0.790	0.038	
	mean FA value	CR-contra	0.396	0.119	0.308	0.065	n.a.	n.a.	0.0764 (vs. CR-R_L)
		WMf-ipsi	0.434	0.083	0.376	0.090	n.a.	n.a.	0.0786
		WMf-contra	0.388	0.103	0.641	0.841	n.a.	n.a.	0.7884
		CR-right (CR-R)	n.a.	n.a.	n.a.	n.a.	0.333	0.048	n.a.
		CR-left (CR-L)	n.a.	n.a.	n.a.	n.a.	0.333	0.052	
	number of fibers	WMf-ipsi	20,392.6	11,720.29	19,629.22	7223.04	n.a.	n.a.	0.6123
		WMf-contra	14,915.27	11,345.83	14,927.22	6190.56	n.a.	n.a.	0.8815

## Data Availability

Data available on request due to restrictions e.g., privacy or ethical.
